# Delayed Diagnosis of Acute Appendicitis in the Third Trimester of Pregnancy: Diagnostic Pitfalls, Multisurgical Management, and a Prolonged Postoperative Course—A Multidisciplinary Case Report

**DOI:** 10.3390/diagnostics15202593

**Published:** 2025-10-14

**Authors:** Gabija Didžiokaitė, Aida Kuznecovaitė, Audrius Šileikis, Virginija Paliulytė

**Affiliations:** 1 Faculty of Medicine, Vilnius University, 01513 Vilnius, Lithuania; aida.kuznecovaite@mf.stud.vu.lt (A.K.); audrius.sileikis@mf.vu.lt (A.Š.); virginija.paliulyte@mf.vu.lt (V.P.); 2 Faculty of Medicine, Institute of Clinical Medicine, Clinic of Gastroenterology, Nephro-Urology and Surgery, Vilnius University, 01513 Vilnius, Lithuania

**Keywords:** acute appendicitis, pregnancy, third trimester, perforated appendix, postoperative complications, intestinal obstruction, eventration, vacuum-assisted closure (VAC)

## Abstract

Background/Objectives: Acute appendicitis is the most common non-obstetric surgical emergency during pregnancy. Diagnosing appendicitis in the third trimester remains especially challenging due to physiological changes that obscure clinical presentation and limit the utility of imaging modalities. These challenges can lead to diagnostic delays, increasing the risk of severe complications for both mother and fetus. Case presentation: We present a complex case of a 36-year-old pregnant woman at 29 + 4 weeks of gestation who developed acute appendicitis with an atypical clinical course. Her initial symptoms were nonspecific and misattributed to gastrointestinal discomfort related to pregnancy. Her condition progressively worsened, leading to an emergency laparoscopic appendectomy. Intraoperative findings confirmed a perforated, necrotic appendix. Postoperatively, she experienced multiple complications, including ileus, wound dehiscence, and complete eventration of the abdominal wall. These required two additional laparotomies and the application of vacuum-assisted closure (VAC) therapy for effective wound management. Despite the severity of maternal complications and the risk of preterm delivery, a multidisciplinary team provided coordinated care. The patient was delivered vaginally at 34 + 4 weeks using vacuum assistance. The neonate, who developed sepsis, was treated in the neonatal intensive care unit and discharged after 24 days. Both mother and child ultimately recovered. Conclusions: This case highlights the diagnostic complexity of appendicitis in late pregnancy and the potential for severe postoperative complications. Prompt surgical intervention, high clinical suspicion, and a multidisciplinary approach are crucial. Moreover, this report adds to the limited literature on the use of VAC therapy for abdominal eventration in pregnancy, demonstrating its feasibility and safety in selected cases.

## 1. Introduction

Appendicitis is one of the most common reasons for non-obstetrical surgical interventions during pregnancy, with an incidence rate between 1:1000 and 1:1500 pregnancies [1]. The majority of symptoms, which would normally be considered as red flags for appendicitis, such as nausea, vomiting, or abdominal pain, which often starts as vague discomfort around the umbilicus and then migrates to the lower quadrant [2,3], in pregnancy may also be regarded as common complaints, caused by the physiological processes during pregnancy [1]. According to various studies, about 0.05–0.2% of pregnant women have acute appendicitis [4]. Although in the general population, the peak incidence of appendicitis is for adolescents and young adults, especially in the 15–19 year age group, the most common age for onset of acute appendicitis in pregnancy is between 20 and 30 years [5,6], with the majority of cases occurring in the second trimester of pregnancy [4]. However, the diagnosis of appendicitis during pregnancy is more complicated due to many physiological changes that occur in the pregnant woman’s body, such as a change in the location of the appendix due to uterine enlargement, physiological leukocytosis, or limitations in radiological examinations due to the risk of radiation [4]. Therefore, the prevalence of acute appendicitis is the highest in the third trimester of pregnancy, when the diagnosis is even more intricate [7]. According to one case-control study conducted in 2024, for 26.6% of pregnant women diagnosed with appendicitis, at the time of the surgery, it had already been perforated [8]. Another study, published in 1999, established that preoperative diagnosis was correct only in 75% of cases, indicating that 25% of cases were potentially diagnosed late or misdiagnosed [9]. Complications such as premature delivery, spontaneous abortion, and stillbirth were reported in the literature, especially when appendicitis occurred in the first or second trimester [9,10]

Ultrasonography and CT scan are considered to be accurate diagnostic modalities for the diagnosis of appendicitis. However, due to the ongoing pregnancy, ultrasonography may not be as informative as expected. Moreover, the option of a CT scan during pregnancy is not always accepted by patients due to the radiation exposure. Therefore, a case of appendicitis during pregnancy poses a real challenge for a timely and accurate diagnosis. Because of a general reluctance to operate unnecessarily on a gravid patient, the diagnosis may be significantly delayed and lead to some serious adverse outcomes. Such factors as rising intra-abdominal pressure and increased susceptibility to pathologic agents during pregnancy also increase the risk of specific postoperative complications, such as eviscerations [11,12]. Delayed diagnosis of this acute condition can lead to more complicated forms of acute appendicitis, which can result in fetal death [6,13].

In this case report, we present a case of acute appendicitis in the third trimester of pregnancy with delayed diagnosis without additional imaging following serious surgical complications and full patient and neonate recovery. Postoperative eventration following appendicitis treatment in pregnancy is exceedingly rare and scarcely documented in medical literature. This case stands out as a unique contribution to the existing clinical knowledge, not only due to the occurrence of eventration in the third trimester but also due to the successful use of vacuum-assisted closure (VAC) therapy in managing the complication.

## 2. Case Report

A 36-year-old pregnant woman was admitted to the admission department at the 30th week (29 weeks + 4 days) of pregnancy with acute abdominal pain localized around the umbilicus and recurrent episodes of diarrhea. The patient informed the attending specialists that she suspected food poisoning as the cause of her symptoms. This was her third pregnancy, with a history of one full-term vaginal delivery assisted by fetal vacuum extraction and one early spontaneous miscarriage.

During the current pregnancy, the patient was diagnosed with antenatal anemia at 23 weeks and was prescribed iron supplementation. Gestational diabetes mellitus was diagnosed at the 24th week and was initially managed with dietary modifications; however, insulin therapy (Ins. Levemir 12 IU subcutaneously in the evening) was initiated at 28 weeks. Allergy to clarithromycin and amoxicillin was reported. No clinical history of chronic diseases or prior surgical interventions was reported.

In the admissions department, the patient received intravenous fluids, analgesics, and drotaverine. She was evaluated by an obstetrician-gynecologist, who found no signs of acute obstetric pathology or preterm labor. No surgical consultation was carried out at that time. Laboratory tests revealed leukocytosis with neutrophilia (WBC 13.60 × 10^9^/L, NEU 83.4%) and a mildly elevated C-reactive protein (CRP 6.16 mg/L), while liver enzymes, kidney function, lipase, and electrolyte levels were within normal limits. Based on the clinical presentation and laboratory findings, the patient was observed overnight and subsequently discharged with recommendations for outpatient follow-up, reduced physical activity, and a bland diet, avoiding spicy and salty foods.

However, one day after discharge, the patient was urgently readmitted to the Abdominal Surgery Unit of a tertiary-level hospital for further evaluation at 29 weeks and 6 days of gestation by her supervising outpatient obstetrician-gynecologist due to persistent abdominal pain and a semi-forced, forward-bent posture, raising suspicion of acute appendicitis. Upon arrival, she reported intensified pain in the right iliac region. Laboratory tests revealed significant leukocytosis and a markedly elevated C-reactive protein level (247.5 mg/L). Physical examination demonstrated signs of an acute abdomen. Although an abdominal ultrasound was performed, the findings were inconclusive. A tubular structure originating from the cecum without peristalsis was observed, extending toward the uterus and not fully visualized. The structure measured up to ~13 mm in diameter, with wall thickness of ~5 mm, and the surrounding tissue appeared infiltrated. A small amount of free fluid was also noted, and the patient reported maximal tenderness at this location. Additional imaging was recommended to confirm the diagnosis.

The pregnant patient underwent an urgent laparoscopic appendectomy, followed by abdominal lavage and drainage. Intraoperatively, the appendix was found in its typical medial position, exhibiting necrosis and perforation. A microbiological culture was obtained from the abdominal cavity, revealing colonization with *E.coli*. Postoperatively, the patient received antibacterial therapy with Cefuroxim 1.5 g intravenously 3 times daily. Following consultation with a clinical pharmacologist, ampicillin (1.0 g intravenously, four times daily) was recommended for further treatment as the bacterial culture obtained showed the highest sensitivity to ampicillin. However, due to the development of an allergic reaction and a dynamic decrease in inflammatory markers, antimicrobial therapy was discontinued within 3 days.

After the surgery, the patient underwent periodic obstetric evaluations, during which fetal well-being was confirmed. Cervical length dynamics were monitored, and at 30 weeks + 2 days of gestation, corticosteroids were administered for fetal lung maturation. At 30 weeks + 6 days, the patient developed regular uterine contractions occurring every five minutes and was subsequently transferred to the Obstetrics Department for labor. However, the contractions subsequently subsided.

The following day, the patient reported cramping abdominal pain exacerbated by fasting and difficulty with defecation. Inflammatory markers were increasing progressively (C-reactive protein [CRP] 38 → 79 mg/L). Abdominal ultrasound, performed at 31 weeks + 1 day of gestation, revealed ascites and small bowel stasis. Over the next two days, the patient experienced worsening pain, followed by episodes of vomiting. Subsequently, the patient developed difficulty eating, and a further elevation of inflammatory markers was observed (CRP 86.1 mg/L). A multidisciplinary team, including obstetricians-gynecologists, abdominal surgeons and clinical pharmacologists, concluded that there was insufficient evidence for immediate surgical intervention. Empirical antibacterial therapy with cefuroxime (3.0 g intravenously, three times daily) was initiated, along with analgesics and laxatives.

**Figure 1:**
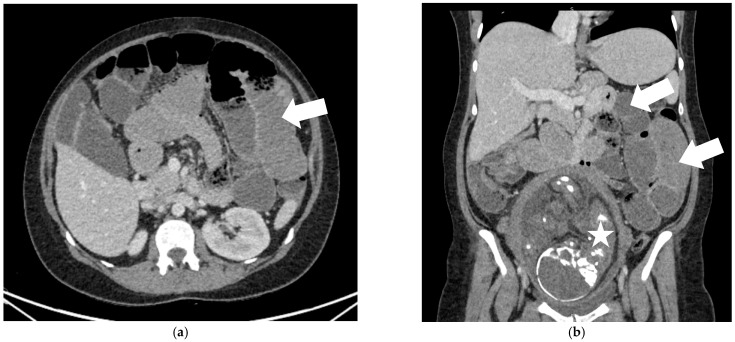
CT scan results 12 days after appendectomy (31 weeks + 4 days) (**a**) Axial CT scan (portovenous phase) showing distended small bowel loops (white arrow)—small bowel obstruction. (**b**) Coronal CT reconstruction (portovenous phase) showing distended small bowel loops (white arrows), fetus (white star).

Despite medical treatment, the patient’s condition continued to deteriorate, with persistent nausea, worsening vomiting, and refractory abdominal pain despite adequate analgesia. At 31 weeks + 4 days, an abdominal computed tomography (CT) scan with contrast was performed, revealing small bowel obstruction due to an ileal segmental blockage, most likely of adhesive origin (Figure 1).

Given these findings, an emergency relaparoscopy was performed, but was subsequently converted into an upper midline laparotomy. During the surgery, the following findings were observed: a highly distended small intestine pushed into the epigastrium, an epiploic appendage of the transverse colon compressing the ileum approximately 40 cm from the ileocecal valve. A strangulating adhesive band was identified in the ileum, but without necrotic changes. A cesarean section was considered due to the potential maternal risks associated with ongoing inflammation and surgical intervention. However, as no signs of fetal distress were observed, it was decided to proceed with laparotomy wound revision and suturing while aiming for a spontaneous vaginal delivery in later stages.

After the second surgery, the woman’s condition improved significantly, with resolution of nausea and vomiting. However, persistent pain at the surgical wound sit remained. At 32 weeks + 2 days of gestation, the patient experienced sudden, severe abdominal pain following a minor oral intake. Examination of the surgical incision revealed suture dehiscence, extensive purulent discharge within the subcutaneous tissue, laparotomy incision eventration, and wound suppuration. An urgent relaparotomy was performed, including wound revision, closure with interrupted sutures, and collection of a pus sample for microbiological culture, revealing colonization with *E.coli* and *Citrobacter amalonaticus.* Intraoperatively, complete eventration along the incision was observed due to suture failure at the right edge of the aponeurosis.

Postoperatively, antibiotic therapy with cefuroxime (3.0 g intravenously, three times daily) was continued. On the first postoperative day, purulent drainage was noted. Given the persistent risk of re-eventration and ongoing wound infection, a multidisciplinary discussion led to the decision to implement vacuum-assisted closure (VAC) therapy for the management of suppurative complications. Under general endotracheal anesthesia, a silver-impregnated sponge (13 × 6 cm) was placed within the wound, and continuous negative pressure therapy at 125 mmHg was applied. Additionally, thromboprophylaxis with enoxaparin (0.3 mL subcutaneously once daily) was initiated, and the patient was advised to wear an abdominal support belt.

**Figure 2:**
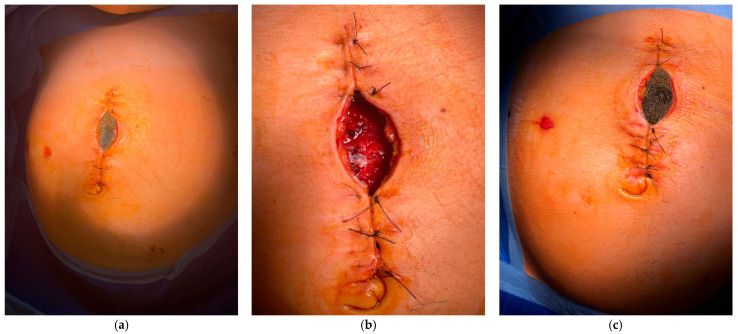
Sequential images of WAC sponge treatment. (**a**) WAC sponge before first removal; (**b**) After first WAC sponge removal; (**c**) Second WAC sponge.

Due to a progressive increase in inflammatory markers (CRP 71.3 → 93.9 mg/L), a clinical pharmacologist was consulted, who recommended escalation of antibiotic therapy from cefuroxime (administered for a total of 11 days) to piperacillin/tazobactam (4.5 g intravenously, four times daily). Due to persistent surgical wound pain, the patient was evaluated by the pain management clinic, where her analgesic regimen was optimized with nonsteroidal anti-inflammatory drugs (intramuscular ketorolac and diclofenac) and an opioid (intramuscular tramadol). VAC sponge replacement was performed at 33 weeks + 2 days under general intubation anaesthesia. Intraoperatively, the sponge was found to be mildly purulent, while the wound bed was covered with granulation tissue (Figure 2). Postoperatively, antibiotic therapy with piperacillin/tazobactam was completed (total duration: 4 days). After the sponge replacement, the patient had no significant complaints, and her general condition remained stable. Obstetric evaluation confirmed fetal well-being.

Recommendations included blood pressure monitoring for three days, biweekly general urinalysis, and a fetal echocardiogram.

The fetal condition was assessed daily using non-stress tests (NST). At 34 weeks of gestation, fetal biometry was performed, indicating appropriate fetal growth for gestational age, normal amniotic fluid volume, no structural abnormalities, and fetoplacental circulation within normal limits.

After the second VAC sponge change at 34 weeks + 4 days, regular contractions started. Vaginal examination confirmed a soft, 4 cm dilated cervix. The patient was transferred to the Obstetrics department for delivery. Due to surgical pathology and the inability to allow prolonged pushing, vacuum-assisted fetal extraction was performed during the second stage of labor.

A male neonate was delivered vaginally, weighing 2720 g, with a length of 47 cm and an Apgar score of 8/8. Despite an initially stable Apgar score, the neonate developed severe respiratory failure, bacterial sepsis, and septic shock. Empiric antibacterial therapy was initiated, and the infant’s condition gradually improved. He was discharged from the hospital after 24 days.

Following delivery, the mother was supervised by abdominal surgeons and obstetrician-gynecologists. Obstetric evaluation in the postoperative period was unremarkable, and lactation suppression was initiated due to the patient’s request.

Eight days postpartum, the VAC sponge was removed under general anesthesia. The surgical wound was noted to be contracted and fully covered with granulation tissue, allowing for secondary closure with a 1–0 prolene suture. The following day, the patient was discharged home in satisfactory condition with recommendations for a restricted activity regimen, avoidance of strenuous physical exertion until complete wound healing, and continuation of iron supplementation for anemia. Analgesics were prescribed as needed for persistent abdominal discomfort.

In total, the patient was hospitalized for 41 days, undergoing seven surgical procedures under general anesthesia and requiring transfer between five different hospital units based on her evolving clinical condition.

## 3. Discussion

Acute appendicitis is the most common non-gynecological and non-obstetric surgical pathology during pregnancy [4]. The primary challenge in diagnosing appendicitis in pregnancy lies in its broad differential diagnosis. While the diagnostic accuracy of appendicitis in the first trimester is approximately 85%, it decreases significantly to only 30% in the third trimester [14]. In this case report, the diagnosis of appendicitis was delayed in the third trimester, leading to unfavourable maternal complications.

Pregnant women often present with symptoms that overlap with physiological changes of pregnancy, complicating timely diagnosis. Hormonal fluctuations can induce nausea, vomiting, and heartburn, while the enlarging uterus can alter pain localization and obscure typical abdominal pain patterns [1]. Additionally, physiological leukocytosis further challenges the reliability of laboratory markers in diagnosing appendicitis [4]. In this case, the patient’s symptoms, including abdominal pain and signs of dyspepsia, could initially be attributed to normal gestational changes, thereby delaying the recognition of a surgical pathology.

The differential diagnosis of acute appendicitis in pregnant women is more complex due to the wide range of conditions that can present with similar symptoms. In general, the differential diagnosis of appendicitis in pregnancy mirrors that in non-pregnant individuals. Conditions that must be considered include Crohn’s disease, complicated cecal diverticulitis, Meckel’s diverticulitis, inflammatory bowel disease, gastroenteritis, right-sided colitis, renal stones, and urinary tract infections [15,16]. Gynecological conditions must also be considered, such as ruptured ovarian cysts, ovarian and fallopian tube cysts, ovarian abscesses, endometriosis, pelvic inflammatory disease, and ectopic pregnancy. However, these diagnoses are quite rare in the third trimester of pregnancy. Additionally, round ligament syndrome should be considered, as it can cause right lower quadrant pain during periods of rapid uterine growth [17,18]. Pregnant women are less likely to exhibit classic signs such as right lower quadrant tenderness, guarding, and rebound tenderness compared to non-pregnant individuals [19,20]. The location of pain may shift with advancing gestational age, and previous cesarean scars can affect the degree of displacement [20].

Ultrasound is the first-line imaging modality for diagnosing acute appendicitis. A retrospective study by Simsek et al., published in 2015, found that only 54% of pregnant women with suspected appendicitis were correctly diagnosed via ultrasound [1]. When ultrasound results are inconclusive, additional imaging techniques such as computed tomography (CT) or magnetic resonance imaging (MRI) are typically employed. Abdominal CT has an accuracy rate of up to 95% in diagnosing acute appendicitis. A primary concern with abdominal CT is radiation exposure. However, the average radiation dose from a routine CT scan is about 4 mSv, which is only slightly higher than the background radiation of approximately 3 mSv [17]. Currently, new CT protocols aim to reduce radiation exposure, further minimizing potential fetal risks. Nevertheless, CT is currently generally recommended only when ultrasound findings are unclear or when MRI is unavailable [21]. A study by Badr et al. demonstrated that MRI successfully visualizes 100% of appendices in pregnant women with acute appendicitis, and the authors suggest that it should be implemented as the initial radiological investigation for suspected appendicitis in pregnant women [22]. In the case we describe, the patient’s altered anatomy due to an enlarged uterus resulted in a suboptimal ultrasound examination.

Delayed diagnosis and treatment of acute appendicitis can lead to complications such as necrotizing or perforated appendicitis. These may in turn result in conditions such as suppurative peritonitis, as observed in the patient discussed in our case report. In cases of complicated perforated appendicitis, urgent surgical intervention is required. Currently, laparoscopic appendectomy is considered the gold standard for surgical treatment due to its short recovery time, lower infection rates, and reduced risk of surgical complications [23,24]. These advantages hold during pregnancy as well, although a higher risk of spontaneous miscarriages is reported. Numerous meta-analyses and systematic reviews consistently report a higher rate of miscarriage after laparoscopic appendectomy compared to open surgery, with the risk nearly doubled [25,26,27]. The reported rate of fetal loss after laparoscopic appendectomy during pregnancy is approximately 6%, which is significantly higher than that associated with open appendectomy. [25,27]. However, the American Society for Gastrointestinal and Endoscopic Surgery (SAGES) affirms that laparoscopic appendectomy can be safely performed at any stage of pregnancy [28]. Similarly, the World Society of Emergency Surgery (WSES) recommends laparoscopic appendectomy over open surgery when indicated [29]. While it was once considered safest to perform laparoscopic appendectomy in the second trimester, recent studies suggest that the trimester of pregnancy does not significantly impact the safety of the procedure for both the mother and the fetus [24,28].

Though antibiotic therapy can be used to treat acute appendicitis, it is only effective for uncomplicated cases. In pregnant women, this approach may also be utilized, but caution is advised regarding the potential risks of antibiotic use during pregnancy, and the effectiveness of this treatment should always be assessed [24]. In Lithuania, preoperative antibiotic therapy, such as with Amoxicillin/Clavulanic acid 1000/200 mg intravenously, is commonly administered to reduce the risk of postoperative infections, though medical management alone is not typically employed for appendicitis [15].

The risk of complications is significantly heightened when the appendix is perforated. The most common post-operative complications following perforated appendicitis include surgical wound infections and suppuration. Peritonitis, resulting from the perforation, is another frequent complication, affecting as many as 50% of patients with a perforated appendix. In pregnancy, uncomplicated acute appendicitis carries a fetal mortality rate of approximately 5%, while this rate increases to 20% in cases of perforated appendicitis [15]. Other studies have reported the risk of perforated appendicitis during pregnancy to be as high as 43–55% [30,31,32]. Additionally, an appendectomy in pregnant women carries the risk of preterm labor. A study by Okcu et al. indicated that 8% of pregnant women who underwent appendectomy during pregnancy experienced preterm birth [33]. In our case, the patient also developed an intestinal obstruction following surgery. To this date, there are no generally agreed guidelines regarding the management of pregnancy in cases of acute appendicitis in the third trimester. While some authors, such as Rahim et al., share case reports where these cases were managed successfully without earlier induction of labor and state that appendicitis during pregnancy is not an indication for delivery per se [34], no consensus exists.

While intestinal obstruction is a known complication after abdominal surgery, it is not commonly reported as a major postoperative issue following appendectomy in pregnancy [35]. Most intestinal obstruction cases are linked to adhesions from prior surgery, which can become problematic as the uterus grows and shifts abdominal organs [36]. However, our patient’s clinical history did not indicate any previous surgeries or pathologies that could have caused adhesions to form in the abdominal cavity.

Wound dehiscence and eventration are rare but serious complications following appendectomy in pregnant women [37]. The risk of wound dehiscence may be heightened in late gestation due to increased abdominal wall tension, as well as by factors such as infection and surgical technique [37]. In severe cases, dehiscence may progress to eventration, particularly in the presence of infection or impaired tissue healing [37,38]. Moreover, the presence of wound infection and suppuration, combined with systemic factors such as gestational diabetes mellitus and anemia, are well-known risk factors for impaired wound healing [39,40]. Although appendectomy itself is generally considered safe in pregnancy and does not significantly increase overall maternal morbidity, the likelihood of postoperative complications such as wound infection and dehiscence is higher in cases of perforated appendicitis or delayed diagnosis [41].

Vacuum-assisted closure (VAC) therapy is a medical technique used to promote wound healing by applying negative pressure to the wound area [42]. VAC therapy works by applying negative pressure to the wound, which promotes rapid wound closure by encouraging perfusion, fibroblast migration, and cell proliferation, removes infected material and excess exudates, reduces localized edema, and draws wound edges closer together [43,44]. VAC therapy is effective in managing high-energy soft tissue injuries, with many patients requiring no further treatment or only a split-thickness skin graft after VAC therapy [45]. It is also used in postoperative settings to manage swelling and drainage, providing a clean and dry wound environment [46]. Schimp et al. in a study involving 27 patients, achieved complete wound healing in 96% of cases, indicating the high success rate of VAC therapy in treating complex wound failures [47].

VAC therapy application during pregnancy, particularly in obstetric emergencies, is a topic of interest due to the unique challenges and potential benefits it presents. VAC therapy has been successfully used in the management of massive puerperal genital hematomas, which are rare but potentially life-threatening complications following childbirth. In a case reported by Yilmaz et al., a pregnant patient developed a large hematoma after a vaginal delivery. The hematoma was initially evacuated, but the wound reopened, prompting the use of VAC therapy. The application of VAC facilitated complete wound closure within ten days, highlighting its effectiveness in managing complex perineal wounds by promoting drainage and granulation tissue formation [48]. Studies have demonstrated that the use of VAC therapy in patients with destructive appendicitis significantly reduces postoperative wound complications. In particular, the incidence of wound complications decreased by three times in patients treated with VAC compared to traditional methods [49].

Eventration, a rare complication following laparotomy, can also be managed using VAC therapy [50]. As described in the case we present, the application of VAC therapy was crucial in managing wound dehiscence and reducing the risk of additional infectious complications. Although literature on VAC therapy in pregnant patients is limited, our case demonstrates its potential benefits in this population. By facilitating wound healing, VAC therapy minimised the need for additional surgical interventions, contributing to a more favourable recovery despite the initial complications.

## 4. Conclusions

Acute appendicitis during pregnancy is the most common non-obstetricemergency requiring surgical intervention. However, its diagnosis is additionally complicated by physiological changes that can mimic normal pregnancy, leading to delayed diagnosis. Early recognition of abdominal pain, especially in the later trimester, is crucial to prevent complications such as perforation and diffuse peritonitis.

In unclear cases when MRI is unavailable, abdominal CT can provide a rapid and accurate diagnosis, enabling timely surgical intervention when necessary. Despite being associated with a slightly higher risk of spontaneous miscarriage, prompt appendectomy, preferably laparoscopic, minimizes general surgical and postoperative complications.

Management requires a collaborative, multidisciplinary approach between obstetricians and surgeons to optimize outcomes for both mother and fetus.

## Data Availability

The original contributions presented in this study are included in the article. Further inquiries can be directed to the corresponding author.
